# Charging Up the Periphery: Glial Ionic Regulation in Sensory Perception

**DOI:** 10.3389/fcell.2021.687732

**Published:** 2021-08-11

**Authors:** Sneha Ray, Aakanksha Singhvi

**Affiliations:** ^1^Division of Basic Sciences, Fred Hutchinson Cancer Research Center, Seattle, WA, United States; ^2^Graduate Program in Neuroscience, University of Washington, Seattle, WA, United States; ^3^Department of Biological Structure, School of Medicine, University of Washington, Seattle, WA, United States

**Keywords:** sense-organs, peripheral glia, sensory perception, ions, non-myelinating

## Abstract

The peripheral nervous system (PNS) receives diverse sensory stimuli from the environment and transmits this information to the central nervous system (CNS) for subsequent processing. Thus, proper functions of cells in peripheral sense organs are a critical gate-keeper to generating appropriate animal sensory behaviors, and indeed their dysfunction tracks sensory deficits, sensorineural disorders, and aging. Like the CNS, the PNS comprises two major cell types, neurons (or sensory cells) and glia (or glia-like supporting neuroepithelial cells). One classic function of PNS glia is to modulate the ionic concentration around associated sensory cells. Here, we review current knowledge of how non-myelinating support cell glia of the PNS regulate the ionic milieu around sensory cell endings across species and systems. Molecular studies reviewed here suggest that, rather than being a passive homeostatic response, glial ionic regulation may in fact actively modulate sensory perception, implying that PNS glia may be active contributors to sensorineural information processing. This is reminiscent of emerging studies suggesting analogous roles for CNS glia in modulating neural circuit processing. We therefore suggest that deeper molecular mechanistic investigations into critical PNS glial functions like ionic regulation are essential to comprehensively understand sensorineural health, disease, and aging.

## Introduction

Glia and neurons comprise the two major cell types of the nervous system of bilaterian animals. Glia of both the central and peripheral nervous systems (CNS and PNS, respectively) are found in close physical and molecular association with cognate neurons or sensory cells. However, despite the near coincident discovery of glia and neurons, the functions of glia remain relatively unknown compared to the depth of our knowledge of neuronal cell biology and physiology. As glia are non-excitable (unlike neurons), analyses of their properties have been historically inaccessible through classical electrophysiological techniques. Recent technological advances in molecular genetics, however, have renewed focus on glia in neuroscience research. In the CNS, glia roles include dictating pioneer axon trajectories, pruning synaptic structures, stabilizing/eliminating synapses, and ionic regulation (see reviews: Chotard and Salecker, 2004; Chung et al., 2013; Allen and Eroglu, 2017). The resulting molecular insights into glia biology have led to a growing appreciation that glia are not passive support cells in the nervous system, but active modulators of neural functions in development, health, and disease.

**FIGURE 1:**
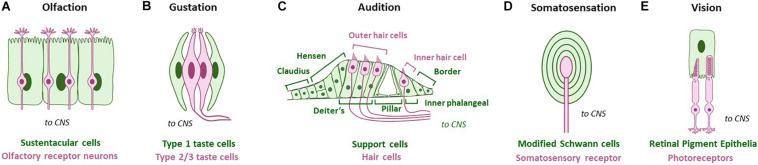
Glia-like support cells at mammalian peripheral sense organs across modalities. Green cells/text are support cells, while magenta cells/text are sensory cells. **(A)** Olfaction: Sustentacular cells appose olfactory receptor neurons. **(B)** Gustation: Type I glia-like cells contact Type II and Type III receptor cells in vertebrate taste buds. **(C)** Audition: Deiter’s support cells interact closely with outer hair cells, and inner phalangeal support cells interact closely with the inner hair cell. Other support cells are also noted. **(D)** Somatosensation: Somatosensory receptors are surrounded by modified Schwann glia cell lamellae. Pacinian corpuscles are shown as example. **(E)** Vision: Glia-like retinal pigment epithelia contact photoreceptor NREs. Adapted using BioRender.com.

Glia associate with neurons at multiple sub-cellular points of contact, including at neuron receptive-endings (NREs). NREs are specialized sub-cellular structures on individual sensory cells or neurons dedicated to receiving input from either the environment or other neurons (Shaham, 2010; Singhvi and Shaham, 2019). In the CNS, this refers to dendritic spines at synapses, where a neuron receives neurotransmitter input from its pre-synaptic neuron partner. While not identical, NREs across the CNS bear significant functional, anatomical and/or molecular similarity. In contrast, each peripheral sense-organ has NREs exquisitely tuned to the sensory modality it transduces. Therefore, PNS NREs vary drastically by sense organ, both anatomically and physiologically. Examples of PNS NREs in mammals include cilia of olfactory neurons, gustatory hairs of taste buds, and microvillar stereocilia of inner hairs cells in the ear (Figure 1).

**FIGURE 2:**
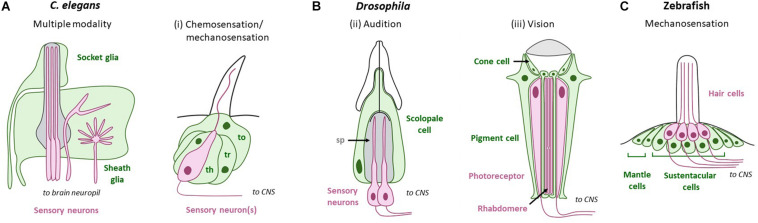
PNS glia across modalities in sense-organs of non-mammal model systems. Green cells/text are support cells, while magenta cells/text are sensory cells. **(A)** *C. elegans* sense-organs comprise socket and sheath glia that ensheath sensory NREs. **(B)** Support cells in *Drosophila*. (i) Chemosensation/mechanosensation: Thecogen (sheath glia; th), trichogen (shaft glia; tr), and tormogen (socket glia; to) associate with neurons in sensory hairs called sensilla. (ii) Audition: Sensory neurons in Johnston’s organ are surrounded by the scolopale space (sp), which is regulated by glia-like scolopale cells. (iii) Vision: *Drosophila* photoreceptor cells are surrounded by two types of glia: pigment cells and cone cells. **(C)** Zebrafish hair cells in the lateral line are organized into neuromasts. Each neuromast houses hair cells intermingled with sustentacular cells at the center, and mantle cells at the periphery. Adapted using BioRender.com.

Nervous system glia come in different subtypes within the CNS and PNS, many of which contact cognate NREs. In mammals, glia of the PNS and CNS have distinct developmental origins, with PNS glia arising from the neural crest, and CNS glia arising from the neural tube (oligodendrocytes and astrocytes) or myeloid lineages (microglia). PNS glia that associate with these are similarly diverse across sensory modalities, presumably tracking PNS-NRE anatomical diversity. Examples of these in mammals include sustentacular cells of the olfactory epithelia and Deiter’s cells in the inner ear (Figure 1). In invertebrates, glia arise from neuroectodermal lineages and are organized with NREs in sense-organs called sensilla. Both NRE and glia biology and anatomy are tailored for the modality sensed (Figure 2).

One classic function of many glial sub-types is to modulate the NRE’s ionic micro-environment. In the mammalian CNS, astrocyte glia regulate extracellular levels of various ions such as potassium (K^+^), sodium (Na^+^), and chloride (Cl^–^), at dendritic spine NREs. Ion buffering at these sites impacts neural circuit dynamics (Simard and Nedergaard, 2004; Olsen et al., 2015). Impairments in ion regulation by CNS glia are implicated in various neurodevelopmental and degenerative disorders including Huntington’s disease, autism and epilepsy (Tong et al., 2014; Verkhratsky, 2019; Song et al., 2020). In striking functional analogy, shared across modalities and with CNS glia, multiple PNS glia sub-types across species also regulate the ionic microenvironment around sensory NREs. As reviewed below, PNS sense organ glia modulate extracellular levels of K^+^, Na^+^, and Cl^–^; as well as calcium (Ca^2+^) ions. This can have functional consequences on sensory cell development and activity, sensory perception and animal behavior. As may be expected then, mutations in PNS glial ion channels and transporters are implicated in many sensory disorders such as deafness and blindness.

Here, we review how PNS glia regulate the neural ionic micro-environment around NREs, across sensory modalities as well as species. This summary focuses extensively on modulation of three ions (K^+^, Na^+^, and Cl^–^) in PNS NRE extracellular space and readers are referred elsewhere for detailed discussion of Ca^2+^ dynamics in PNS neural cells (Newman and Zahs, 1998; Anselmi et al., 2008; Hegg et al., 2009). We collate this in the context of sense-organ neuroanatomy and discuss how ionic modulation by PNS glia can impact sensory NRE shape, neural activity, and sensory processing. Finally, we highlight gaps in knowledge in each modality, and conclude with evaluating evolutionary conservation across species and systems. We suggest that instead of a passive support function, glial ionic regulation of sensory cell/NRE milieu can be viewed as an active modifier of animal sensory perception.

## Regulation of Sensory Cell Ionic Milieu by PNS Glia

### Olfaction

Olfaction is the perception of volatile external chemical stimuli through either the nose (vertebrates) or chemosensory sensilla (invertebrates). In both groups, glia-like support cells adjacent to primary chemosensory sensory NREs influence sensation by regulating local levels of K^+^, Na^+^, and Cl^–^. Besides ionic regulation, additional functions ascribed to chemosensory sense-organ glia include metabolic support of ORNs and odorant modification, buffering, and clearance (Heydel et al., 2013; Larter et al., 2016).

#### Mammals

Mammals perceive volatile chemicals through specialized odorant receptors on the ciliary NREs of olfactory receptor neurons (ORNs) in the main olfactory nasal epithelium (Buck, 2004). Embedded along with ORNs within this epithelium are support glia called sustentacular cells (SCs; Figure 1A). SC glia form a tightly packed columnar monolayer on the apical surface of the olfactory epithelium. They have microvilli that access the lumen of the nasal cavity where ORNs detect odorants through either ciliated or microvilli NREs, each bearing distinct receptors (Elsaesser and Paysan, 2007; Chen et al., 2014). The mammalian vomeronasal organ, which detects pheromones, has a similar anatomy. To our knowledge, whether all support glia across the olfactory epithelia, associated with either ciliary or microvillar ORNs, are molecularly and functionally analogous remains unknown.

Sustentacular cell glia in mice generate voltage gated Na^+^ and K^+^ currents, and have outwardly rectifying leak conductance (Vogalis et al., 2005). Voltage gated Na^+^ channels are inactive at SC resting potentials, and their functions in sensory perception await inquiry. The voltage-gated K^+^ current is largely generated by charybdotoxin-sensitive calcium (Ca^2+^)-activated BK channels. These channels are active at rest and densely localized on the apical surfaces of SC glia. The polarized localization of these channels suggests vectoral transport of K^+^ ions in SC glia; however, the existence of such directional flow has yet to be established. The leak conductance in SC glia is equally permeable to Cs^+^, K^+^, and Na^+^ and also likely passes anions such as Cl^–^ and F^–^. Such broad permeability suggests that the leak current is generated in part by the opening of gap junction channels. In support of this model, application of the connexon-blocker 18β-GA causes a ∼3–4× increase in the resting membrane potential of sustentacular cells in the neonate (Vogalis et al., 2005). How electrical coupling between groups of SC glia impacts neuron activity or sensory animal behaviors remains unknown.

The vomeronasal organ (VNO) detects mammalian pheromones. Ion substitution patch-clamp studies in neonate mice found that VNO-SC membranes have high resting permeabilities to K^+^, Na^+^, and Cl^–^ (Ghiaroni et al., 2003). Since pheromone-laden fluids like rodent urine have high concentrations of K^+^, Na^+^, and Cl^–^, this suggests two plausible mechanisms by which SCs could modify pheromone-sensing. First, spatial buffering of K^+^, Na^+^, and Cl^–^ ions by support cells could alter VNO sensory neuron membrane excitability. Second, ionic modification of the pheromone-containing fluid could impact the biophysical properties of the pheromone molecules themselves or their binding to cognate receptors.

Neonatal VNO SCs also display voltage-gated K^+^ (outward) and Na^+^ (inward) currents (Ghiaroni et al., 2003). The uniformity of voltage gated currents across cells indicates that VNO support cells may be a homogeneous population. Voltage gated K^+^ currents in VNO-SCs are similar to delayed rectifier types (K_*DR*_) described in other glia, with some key pharmacological and electrophysiological differences. While K_*DR*_ channels in astrocytes and Schwann cells are only moderately sensitive to the non-selective K^+^ channel blocker TEA, VNO-SC K_*DR*_ channels show high sensitivity to TEA. Further, K_*DR*_ channels in astrocytes are equally sensitive to TEA and 4-AP, which blocks KCNA family of voltage-activated K^+^ channels. On the other hand, VNO support cell K_*DR*_ channels display a lower sensitivity to 4-AP compared to TEA. VNO support cell K^+^ currents are also unusually noisy, compared to K_*DR*_ currents in other cells, and are not Ca^2+^-dependent, unlike charybdotoxin-sensitive BK channels in olfactory SCs. In contrast, Na^+^ channels in VNO support cells resemble Na^+^ channels of CNS protoplasmic astrocytes in electrophysiological and pharmacological properties, with both having steady-state inactivation and low sensitivity to the voltage-gated Na^+^ channel blocker TTX (Ghiaroni et al., 2003). The exact identity and functional importance of VNO-SC K^+^ channels and Na^+^ channels have yet to be established. Spatial buffering, or the redistribution of K^+^ ions in the extracellular space, may involve both resting and voltage-gated K^+^ channels. Such ionic adjustment of the VNO lumen can impact VNO sensory neuron resting membrane potential and consequent activity. Importantly, investigations in mature VNO support cells are necessary to discriminate between developmental and adult ionic modulation.

#### Drosophila

Flies use special innervated hairs, or sensilla, to sense external chemicals. Each sensillum houses one or more sensory neuron(s), along with stereotyped glia-like support cells called shaft glia (trichogen), sheath glia (thecogen), and one or two socket glia (tormogen) (Hartenstein, 1988; Figure 2Bi). The role of these glia in ionic regulation is unclear.

#### C. elegans

The invertebrate model, *C. elegans*, has 50 ectoderm-derived glia and 6 mesoderm-lineage glia-like cells. All 50 ectoderm-lineage glia physically approximate sensory neurons within sensilla and can be sub-typed as sheath or socket glia, similar to *Drosophila*. The *C. elegans* chemosensory system relies on three sense-organs: the amphid, inner labial organ, and phasmid, with the first two located in the animal nose tip and the last in the tail. Of these, the amphid sensillum is the primary and best studied sense-organ of the animal. This sensory structure consists of the amphid sheath glia (AMsh) and amphid socket glial cell (AMso), which both associate with 12 sensory NREs, including those of 11 chemosensory neurons (Ward et al., 1975; Perkins et al., 1986; Singhvi and Shaham, 2019; Figure 2A). Glial roles in the phasmid and inner labial organ still await investigation.

AMsh glia modulate NRE shape and/or functions of its associated chemosensory neurons (Bacaj et al., 2008; Wang et al., 2008, 2012; Singhvi et al., 2016). However, the specific glia-neuron interactions underlying this are largely unknown, barring a few molecules. In the context of ionic regulation, AMsh glia expression of the Degenerin/epithelial (DEG/ENaC) Na^+^-selective channel ACD-1 has been shown to support function of the odor-sensing AWC neuron. Wildtype animals display chemotaxis toward and changes in AWC intracellular Ca^2+^ levels upon exposure to the AWC-sensed odorant isoamyl alcohol (IAA). Animals with a loss-of-function allele of AMsh *acd-1* or a hypomorphic allele of neuronal *tax-2*, a subunit of the cGMP-gated channel required for chemosensation, show intact chemotaxis and appropriate AWC Ca^2+^ responses to IAA. Double mutations in both AMsh *acd-1* and neuronal *tax-2*, however, impairs IAA chemotaxis and AWC Ca^2+^ responses to low levels of IAA. Knockout of AMsh *acd-1* further exacerbates AWC chemotaxis defects caused by mutations in the G_*i*_ protein ODR-3 and the guanylate cyclase protein DAF-11, which are proposed to regulate the activity of TAX-2 channels. Interestingly, exogenous depolarization of AWC neurons in *acd-1; tax-2* double mutants rescue these sensory deficits to IAA. These studies suggest that glial Na^+^ transport may contribute either directly or indirectly to chemosensation, possibly by regulating basal neuron excitability. This effect of AMsh ACD-1 also appears to be odor specific, as *acd-1* knockout does not exacerbate chemotaxis defects of hypo-morphic *tax-2* mutants to octanol or trimethylthiazole.

AMsh glia also expresses the Cl^–^ channel CLH-1, which is permeable to Cl^–^ and HCO_3_^–^. This channel has been shown to regulate AMsh intracellular pH via HCO_3_^–^ flux through CLH-1. Interestingly, glial ACD-1 currents are strongly inhibited by both extra- and intracellular acidification, suggesting that pH modulation by CLH-1 may play a role in chemosensation (Grant et al., 2015). The direct role of this channel on sensation, however, has yet to be investigated.

#### Other

The olfactory epithelium of frogs and salamanders bear similar anatomy to that in mammals (see Figure 1A), and SCs in both organisms are thought to play a role in K^+^ buffering. Patch clamp studies in frog and salamander olfactory SCs describe high K^+^ permeabilities. In the frog, SC K^+^ currents are mediated by Ca^2+^-activated BK channels, similar to mammalian olfactory SCs, suggesting evolutionary conservation. Frog and salamander SCs further exhibit membrane depolarization after odorant stimulation, likely due to the influx of excess extracellular K^+^ following ORN activation (Trotier and MacLeod, 1986; Trotier, 1998). Whole cell recordings with a gap junction blocker in frog SCs reveals electrical coupling between olfactory support cells, which could facilitate the clearance of K^+^ ions released by ORNs (Trotier, 1998). Electrophysiological studies suggest that K^+^ buffering in salamander SCs occurs in hundreds of milliseconds, which parallels the timescale of mouse ORN activation (Trotier and MacLeod, 1986). This suggests that SCs are well poised to regulate ORN sensitivity to odors in real time. Whether and how K^+^ buffering in frog or salamander SCs affects chemosensation, however, has yet to be formally investigated.

### Gustation

Taste is the percept of non-volatile external stimuli including ions. Roles of glia in gustation are poorly understood across systems.

#### Mammals

Taste sensation in mammals begins in taste buds, onion bulb-like structures in the tongue (Figure 1B). Each taste bud consists of 50–100 elongated neuroepithelial cells that are sub-categorized as Types 1–3. Type 2 and Type 3 cells are taste receptor cells that sample tastants through taste receptors housed on primary cilia NREs (Yarmolinsky et al., 2009; Roper and Chaudhari, 2017). Type 1 cells, on the other hand, exhibit glia-like functions both chemically and structurally. Similar to CNS glia, Type I cells express transporters that are required to eliminate extracellular neurotransmitters, including the glutamate-aspartate transporter GLAST. Further, similar to the close interactions of CNS glia and neurons, Type I cells have cytoplasmic extensions that ensheath other taste bud cells (Lawton et al., 2000; Bartel et al., 2006; Roper and Chaudhari, 2017). Taste bud glia remain molecularly enigmatic.

Type I taste bud glia-like cells display a small voltage-dependent outward K^+^ current and express the inward-rectified K^+^ channel ROMK (Medler et al., 2003; Romanov and Kolesnikov, 2006; Dvoryanchikov et al., 2009). This suggests that these cells may spatially buffer K^+^ in the taste bud, which could alter Type II and Type III taste cell excitability. They are also the only taste bud cells to express amiloride-sensitive ENaC Na^+^ channels. Behavioral studies in rodents show that amiloride decreases taste perception of NaCl, suggesting that amiloride-sensitive ENaC channels play a crucial role in taste transduction of salt. Type I cell expression of these channels implicates a role of these glia-like cells in salt detection (Vandenbeuch et al., 2008). Further studies are required to illuminate the precise role of glial ENaC Na^+^ channels in salt sensing.

#### C. elegans

In addition to olfaction, the AMsh glial DEG/ENaC Na^+^ channel ACD-1 is also required to sense water-soluble tastants, such as NaCl, acidic solutions, and lysine acetate.

Similar to IAA chemosensation (see above), AMsh *acd-1* knockout exacerbates the reduced chemotaxis of hypomorphic *tax-2* mutants to NaCl. Acid avoidance and chemotaxis to the amino acid lysine requires the neuronal DEG/ENaC channel DEG-1. AMsh *acd-1* knockout further exacerbates the impaired acid avoidance and lysine chemotaxis of *deg-1* mutants. Similar to olfaction, these findings suggest that AMsh ACD-1 plays a role in setting chemosensory neuron excitability. As the AMsh HCO_3_^–^ channel CLH-1 may affect ACD-1 activity (see above), glial regulation of HCO_3_^–^ may additionally play a role in taste sensation (Wang et al., 2008; Wang et al., 2012). The precise mechanistic role of ACD-1 and CLH-1 in sensing water-soluble tastants awaits further investigation.

### Auditory and Vestibular Sensation

The auditory system is exteroceptive and mediates sound perception, while the vestibular system is proprioceptive and concerned with maintaining body equilibrium and orientation in space. Since these systems are anatomically and functionally related, they are discussed together here. Glia-like support cells adjacent to peripheral auditory and vestibular sensory structures influence sensation by regulating local levels of K^+^, Na^+^, and Cl^–^. Besides ion regulation, auditory/vestibular support cells modulate the extracellular matrix to support sensory epithelial integrity, form new hair cells upon injury, and engulf synaptic terminals.

#### Mammals

Hearing and balance in mammals is mediated by modified epithelial cells called hair cells in the cochlea and vestibular system of the ear, respectively. Cochlear hair cells are activated by sound waves, and vestibular hair cells by head movements and gravitational force (Corey, 2009; Reichenbach and Hudspeth, 2014). The cochlea is anatomically divided into three compartments, the scala media, scala tympani, and scala vestibuli. The developing cochlea also contains a transient epithelial structure called Kolliker’s organ, which comprises columnar-shaped supporting cells that differentiate into auditory sensory cells. Each adult hair cell NRE comprises ∼100 actin-based stereocilia (microvilli) and a single cilium termed the kinocilium. Cochlear hair cells are surrounded by various glial cell-types that are location-specific, such as inner phalangeal cells, Deiter’s cells, and cells of Hensen’s (Figure 1C). In contrast, the vestibular system anatomically has a more homogeneous group of support cells (Wan et al., 2013).

The neural physiology of hair cells differs from that of most nervous system cells. While most neurons fire action potentials by influx of Na^+^, hair cells rely on K^+^ entry for neuronal activation. This requires K^+^ depolarization, which in turn requires that hair cell NREs are bathed in the high K^+^/low Na^+^ ionic environment of the scala media endolymph. Endolymph ion composition uniquely resembles intracellular fluid, while the low K^+^/high Na^+^ perilymph of the scala tympani and scala vestibuli are typical of extracellular spaces. Support cells help keep endolymph and perilymph compositions distinct by serving as a neuroepithelial barrier between their respective compartments. To do so, support cells make extensive bicellular and tricellular tight junctions (TJs) with hair cells. Mutations in junctional proteins concentrated at support cell-hair cell TJs, including claudin 14, tricellulin, and angulin-2/ILDR1, cause deafness (Wilcox et al., 2001; Riazuddin et al., 2006; Kitajiri and Katsuno, 2016). Therefore, the composition of this endolymph, and thereby auditory perception, requires the surrounding glia.

Upon sensory transduction, K^+^ flows passively into hair cells from the endolymph and exits through basolateral KCNQ4 channels into the perilymph. Radiotracer and molecular genetic studies show that endolymph fluid derives from the surrounding perilymph, rather than the blood. This suggests that K^+^ is recycled from the perilymph back to the endolymph. Two support-cell dependent pathways, the lateral and medial transport routes (see below), are thought to mediate K^+^ recycling. Mutations in support cell proteins involved in K^+^ recycling lead to hearing loss, highlighting the importance of this process to auditory perception (Zdebik et al., 2009).

The lateral transport route is the canonical pathway underlying K^+^ recycling from the perilymph to the endolymph. Here, the stria vascularis of the cochlea or dark cells of the vestibular system secrete K^+^ into the endolymph. Three non-exclusive theoretical models describe how perilymph K^+^ gets to the stria vascularis. We summarize these briefly here, and refer readers to Zdebik et al. (2009) for a deeper review. *Model A:* Glia-like Deiter’s and inner phalangeal cells uptake K^+^ from the basal poles of hair cells using K^+^/Cl^–^ co-transporters (KCCs) and relay K^+^ back to the stria vascularis through a system of gap junctions and transporters. Consistent with this, Deiter’s glia-like cells express the K/Cl co-transporters KCC-4 and KCC-3, and inner phalangeal support cells express KCC-3 (Boettger et al., 2003). Knockout of either *kcc-3* or *kcc4* results in progressive hearing loss (Boettger et al., 2003). In agreement with their role in support glia, auditory defects are observed in ubiquitous *kcc-3* knockout animals, but not in neuron-specific *kcc-3* knockout animals (Shekarabi et al., 2012). As further support for this model, audio-vestibular support cells are electrically coupled by gap junctions made predominantly of connexin-26 (Cx26) and connexin-30 (Cx30) subunits (Nadol et al., 1976; Hama and Saito, 1977; Kikuchi et al., 1994; Zhao et al., 2006). Cx26 and Cx30 are mutated in hereditary forms of deafness, and knockout mouse models replicate this phenotype. Interestingly, targeted ablation of Cx26 in support cells and flanking epithelial cells alone can cause hearing impairment (Cohen-Salmon et al., 2002; Stong et al., 2006). *Model B:* Glial support cells use KCCs to relay K^+^ from the basal poles of hair cells to the scala tympani. From here, K+ travels through the open perilymph space to the stria vascularis for secretion into the endolymph. This model is consistent with the *kcc-3* and *kcc-4* gene expression and mutant studies above. In further support, the perilymph of the scala tympani contains standing currents that are altered upon acoustic stimulation (Zidanic and Brownell, 1990). To note, however, standing currents can also exist if K^+^ is directly shuttled into the scala tympani without support cells. *Model C:* K^+^ enters glial support cells through P2X purinergic signaling and is shuttled through gap junctions or via the perilymph back to the stria vasicularis. Consistent with this, sub/micromolar levels of ATP evoke a K^+^ dependent inward current in cochlear glia, including Deiters, Pillar, Hensen, and Claudius cells. This current tracks [ATP] and is abolished in the absence of ATP or with P2X receptor antagonists (Zhu and Zhao, 2010). Since gap channel hemichannels release ATP, support glia gap junctions may themselves trigger K^+^ intake (Zhao et al., 2005).

In the putative medial K^+^ recycling pathway, K^+^ effluxed from inner hair cells travels through border support cells and inner ear epithelial cells to return directly to the scala media. This is supported by structural and immunohistochemical evidence. Namely, border and epithelial cells have morphological and histochemical features similar to Hensen and Claudius cells of the lateral transport route (Spicer and Schulte, 1998). These cells also have gap junctions that might allow transcellular transport of K^+^ ions (Spicer and Schulte, 1998). However, direct evidence of this route has yet to be demonstrated. If the medial route is a canonical K^+^ recycling pathway, this would suggest that border support cells of the inner ear mediate K^+^ levels in the auditory NRE extracellular milieu, with possibly consequences on hearing.

Maintenance of high K^+^ in the endolymph necessitates a counterbalance of Na^+^ absorption. Na^+^ ion imbalance in the endolymph causes blebbing of the inner hair cell NREs (Kim and Marcus, 2011). Na^+^ and K^+^ endolymph concentration are maintained by proteins like Na^+^/K^+^ ATPase, expressed by both Dieter’s cells and the inner phalangeal cells of the organ of Corti, among other inner ear cells. Mutations in this ATPase pump are associated with Meniere’s disease, an inner ear condition characterized by dizzy spells (vertigo) and hearing loss (Stephenson et al., 2021). Caudius’ and Deiter’s support cell glia also express all three subunits of ENaC channels (Gründer et al., 2001). Interestingly, mutations in the transmembrane serine protease TMPRSS3, thought to regulate ENaC channels, is implicated in non-syndromic autosomal recessive deafness (DFNA8/10) (Guipponi et al., 2002).

Ionic regulation by support cells is also important for hair cell development. It can stimulate the spontaneous firing of auditory neurons before the onset of hearing, a process required for auditory neuron survival and maturation and synaptic refinement. Specifically, support cells in the transient Kolliker’s organ of the developing mammalian cochlea spontaneously release ATP, likely through connexin hemichannels, stimulating purinergic P2RY1 support cell auto-receptors (Tritsch et al., 2007). Activation of purinergic auto-receptors opens support cell TMEM16 Ca^2+^-activated Cl^–^ channels, triggering osmotic cell shrinkage, Cl^–^ efflux, and concurrent K^+^ efflux (Wang et al., 2015; Babola et al., 2020). K^+^ efflux from support cells causes transient depolarization of IHCs near ATP release sites and triggers action potential bursts in primary auditory neurons. Osmotic shrinkage of support cells also increases the extracellular space and speed of K^+^ redistribution. Thus, by modulating Cl^–^ flux, support cells can both initiate spontaneous hair cell activity and regulate hair cell excitability by controlling the volume of the extracellular space.

#### Drosophila

Fly hearing is mediated by sensilla in the chordotonal Johnston’s organ (JO) in the antenna of the animal (Figure 2Bii), which detect air vibrations. The JO is comprised of an array of ∼250 auditory units called scolopidia that house two to three sensory neurons with cilia NREs, and several glia-like support cells. The principle support cell, scolopale, encloses the neuronal dendrite NRE in fluid-filled lumen called the scolopale space. Similar to the vertebrate cochlear endolymph, this fluid is believed to rich in K^+^ ions, although the composition has not yet been analyzed (Eberl and Boekhoff-Falk, 2007).

In the JO, the ATP∝ Na^+^/K^+^ ATPase subunit is enriched in scolopale cells, and knockdown in the entire JO or specifically in scolopale cells results in animal deafness. The Na^+^/K^+^ ATPase extrudes Na^+^ out and K^+^ in in a 3:2 ratio. Knockdown of the nrv2 β Na^+^/K^+^ ATPase subunit, expressed specifically in scolopale cells in the JO, similarly results in almost complete deafness (Roy et al., 2013). Thus, ion regulation by scolopale support cell glia may be crucial for proper auditory transduction and setting sensory neuron excitability, although this has not been formally demonstrated.

#### Other

The zebrafish, *Danio rerio*, senses mechanical vibrations via two sensory systems. One is the otic placode-derived inner ear, which resembles mammalian ear except that it lacks cochlea (Popper and Fay, 1993; Nicolson, 2017; Pickett and Raible, 2019). In addition, mechanosensitive hair cells of the lateral line sense movement, vibration, and water pressure. This is critical for the animals to orient, school socially, and detect predators. Lateral line hair cells bear morphologic and genetic resemblance to those of the vertebrate inner ear, and also have NREs composed of stereocilia and a kinocilium that mediate hair cell activation (Pinto-Teixeira et al., 2015; Nicolson, 2017). Hair cells are organized into a collection of neuromasts spaced along the body of the animal. Each neuromast consists of roughly 60 cells organized in a circular epithelium: 16–20 mechanosensory hair cells and ∼30 glia-like sustentacular cells intermingle at the center of the organ, while ∼10 glia-like mantle cells occupy the periphery (Figure 2C). Within the neuromast, support cell gap junctions maintain low K^+^ levels in associated hair cells, likely through K^+^ buffering. Pharmacologically blocking gap junctions in support cells drastically reduces presynaptic Ca^2+^ activity in hair cells, with impact on sensory stimuli encoding (Zhang et al., 2018). Molecular mechanisms regulating this function are not well-elucidated.

### Somatosensation

Somatosensation includes processing of information received at the skin/cuticle, and includes sub-modalities such as mechanoreception, thermosensation, and nociception. Together, these enable the animal to sense touch, pressure, stretch, heat, cold, and pain (Abraira and Ginty, 2013). The role of glia on somatosensation remains largely a mystery.

#### Mammals

A subset of somatosensory NREs in the vertebrate skin are encapsulated by lamellae of modified glial Schwann cells (Figure 1D). These structures include Meissner’s corpuscles (which sense pressure), Pacinian corpuscles (which sense vibration), and Ruffini’s corpuscles (which sense skin stretch). Both physiological and biophysical studies suggest that this lamellar encapsulation contributes to somatosensory perception (Cobo et al., 2021).

Ion channels are present in the modified Schwann cells of somatosensory receptors. Histological studies have shown that the DEG/ENaC Na^+^ channel ASIC2 and ENaCβ subunit are present in lamellar cells of Pacinian corpuscles and terminal Schwann cells of Ruffini corpuscles, respectively (Hitomi et al., 2009; Montaño et al., 2009; Calavia et al., 2010). Since DEG/ENaC channels have been implicated in touch sensation in many systems (Ben-Shahar, 2011), this raises the possibility that Schwann cells associated with these encapsulated receptors may play a mechanosensory role. In addition, voltage-gated Na^+^ channels have also been identified by immunocytochemistry in Pacinian corpuscle lamellae, suggesting that these may also contribute to mechano-transduction (Pawson et al., 2000). Similarly, immunohistochemistry also reveals the presence of the polymodal Ca^2+^ permeant channel TRPV4 (a known mechano-sensor itself) in the lamellar cells of human Meissner corpuscles (Alonso-González et al., 2017). However, the contribution of TRPV4 channels and lamellar glia in pressure sensation has not yet been established.

#### C. elegans

The OLQ and IL1 glial sheath and socket cells, which associate with OLQ and IL1 mechanosensory neurons, express the DEG/ENaC subunits DELM-1 and DELM-2. These ion channels act cell-autonomously in the OLQ and IL1 glial socket cells to drive sensitivity to mechanical stimulus and nose touch and foraging behaviors. Indeed, animals lacking DELM-1 exhibit defects in setting basal OLQ and IL1 neuron excitability (Han et al., 2013). This shows direct evidence of glial ion channels mediating mechano-sensation.

*C. elegans* thermosensation is mediated by the temperature-sensing AFD neuron. AMsh glia directly regulate thermosensation by modulating the microvilli NRE shape of AFD. One mechanism by which AMsh glia control AFD microvilli NRE shape is using the glial K/Cl transporter, KCC-3, which localizes specifically around the AFD NREs. Molecularly, Cl^–^ extruded by the AMsh glia directly inhibits the GCY-8 receptor guanylyl cyclase located on AFD NREs. cGMP generated by GCY-8 antagonizes the actin regulator WSP-1/NWASP which drives actin assembly and polymerization (Singhvi et al., 2016). Thus, inhibition of GCY-8 by AMsh glial KCC-3 reduces AFD cGMP levels, allowing WSP-1/NWASP to properly shape AFD NRE microvilli. *kcc-3* mutants have short AFD microvilli and, consequently, an inability to perform wildtype thermotaxis behaviors. These findings describe a precise molecular mechanism by which ionic regulation by glia mediates animal sensation. Whether other glial ion channels and transporters also controls thermosensation remains uninvestigated.

Further, recent work from our laboratory has found that AMsh glia engulf fragments of AFD NRE in an activity dependent manner (Raiders et al., 2021a). Altering this glial pruning activity can alter NRE shape and associated animal behaviors post embryonic development, similar to ionic modulation by glial KCC-3. How glial ionic regulators like KCC-3 cooperate with other glial regulatory pathways like pruning of NREs is not clear in any setting.

#### Other Animals

In the spider (*Cupiennius salei*) mechanosensory organ, Na^+^ channels are expressed at similar levels in both neurons and their surrounding glial cells, as quantified by immunofluorescence (Seyfarth et al., 1995). These glia may regulate Na^+^ levels in mechanosensory organs to regulate sensation.

### Vision

Vision is the transduction of light stimuli through photosensitive cells. Glia-like support cells adjacent to light-sensing NREs have been shown regulate local levels of K^+^, Na^+^, and Cl^–^ ions to affect vision. Besides ionic regulation, additional functions of sense-organ glia include regeneration of visual pigments retinal and phagocytosis of photoreceptor NREs.

#### Mammals

The mammalian retina develops from the neural tube rather than the neural crest, making vision the only vertebrate sensory modality that is technically CNS and not PNS. However, because the retina is a sense organ, we include the role of glial ionic modulation in mammalian visual sensation for completeness.

Mammals sense light using photoreceptor cells called rods and cones, whose NREs (outer-segments) are modified cilia. Retinal pigment epithelia (RPE), a polarized monolayer of glia-like support cells, form the outermost layer of the retina and physically approximate photoreceptor NREs. Microvilli extend from the apical surface of RPE cells to envelop the outer segments of both rods and cones (Reichenbach and Bringmann, 2020; Figure 1E). RPE glia-like cells transport ions and water from the apical subretinal space (proximal to photoreceptor NRE) to the basolateral vasculature, or choroid. Tight junctions between RPE ensure a barrier between these spaces. Below we discuss ions individually for simplicity, but also note that each ionic current is also influenced by the concentration and conductance of other ions.

#### Na^+^

Unlike most epithelia, RPE localize Na^+^/K^+^ ATPases to their apical membranes for transepithelial transport. This establishes a Na^+^ gradient that facilitates bicarbonate uptake via a Na^+^-bicarbonate transporter, and uptake of K^+^ and Cl^–^ through the NKCC cotransporter (Strauss, 2005). Further, unstimulated photoreceptors passively intake Na^+^ ions through open cyclic-nucleotide gated channels. This “dark current” establishes the depolarized resting potential needed for vision. Apically localized RPE Na^+^/K^+^ ATPase in the RPE help set the sub-retinal Na^+^ concentration needed to drive these currents, a critical requisite for visual perception (Sparrow et al., 2010).

#### K^+^

K^+^ enters RPE apically through either the Na^+^/K+ ATPase or NKCC. From the RPE, K^+^ ions can leave the cell through either the apical, or basolateral membranes. In dark, photoreceptors intake Na^+^ and extrude K^+^ (see above). The consequent high K^+^ levels in subretinal space promote K^+^ uptake apically and release to the basolateral choroid. Higher basolateral K^+^ conductance allows for net K^+^ transport from photoreceptor space to basolateral vasculature. The channels/transporters driving basolateral conductance await molecular identification, but candidates include the large-conductance Ca^2+^-dependent K^+^ channels and the M-type K^+^ channel (Strauss, 2005). Upon light-stimulation, photoreceptor dark current drops, which reduces subretinal K^+^ levels. This hyperpolarizes the apical RPE membrane, which activates apical Kir7.1 inward rectifying K^+^ channels. Activation of Kir7.1 channels drive K^+^ efflux out of the apical membrane, which replenish subretinal K^+^ levels. Such RPE K^+^ buffering crucial for the fast-occurring changes during visual transduction and maintaining photoreceptor excitability for repeated activation (Strauss, 2005; Sparrow et al., 2010). While mechanisms by which RPE regulate Kir7.1 remains unclear, this likely impacts visual function.

#### Cl^–^

In addition to K^+^, the apical NKCC cotransporter also facilitates entry of Cl^–^ into the RPE. Intracellular Cl^–^ exits the cell through basolateral Ca^2+^-dependent Cl^–^ channels including CLC-2, and the cystic fibrosis transmembrane conductance regulator (CFTR) (Strauss, 2005). Transgenic mice lacking ClC-2 channels show no RPE transepithelial potential and exhibit retinal degeneration comparable to retinitis pigmentosa (Bösl et al., 2001). Best’s vitelliform macular degeneration, in which RPE degeneration causes retinal degeneration, is also associated with defective Cl^–^ transport. The leading diagnostic in this case is a reduction in the light peak-to-dark ratio in an electro-oculogram and extracellular fluid filled lesions, both indicative of decreased RPE basolateral Cl^–^ transport (Xiao et al., 2010). However, mechanistic insights into these regulators in health or disease remains poorly understood.

Reduced RPE transepithelial ion transport is associated with impaired visual function. For example, macular edema is the buildup of fluid in the center of the retina, likely caused by damage to the RPE/endothelium mediated blood/retina barrier. It is successfully treated with carbonic anhydrase inhibitors. These reduce RPE intracellular bicarbonate concentration by reducing uptake of Cl^–^ by the basolateral Cl^–^/bicarbonate exchanger and driving its release baso-laterally instead. This Cl^–^ extrusion drives water transport into the choroid, eliminating the edematous fluid. Thus, ionic transport by RPE cells is critical for retinal health.

#### Drosophila

The *Drosophila* compound eye consists of units called ommatidia. Each ommatidium houses 8 core photoreceptor neurons (R-cells) that sense light using microvilli NREs called rhabdomeres. These R-cell bundles are additionally surrounded by four support cells, called cone cells, and two pigment cells (Figure 2Biii). Cone cells express sense-organ glial signature genes (eg. *pax-2, prox-1*, and *olig-1*) and may serve glial functions like energy support and sustaining photoreceptor neurotransmission in the fly retina (Chotard and Salecker, 2007; Charlton-Perkins et al., 2017). Dark adapted flies exhibit strong photoreceptor depolarization in response to light, as measured by electroretinograms. Genetic knockdown of the ATP-alpha, nrv2, or nrv3 subunit of the Na^+^/K^+^ pump in cone cells significantly reduced photoreceptor responses to light. Knock-down of cone cell inward rectifying Irk2 K^+^ channel similarly reduced photoreceptor responses (Charlton-Perkins et al., 2017). Thus, ion regulation by retinal glia are crucial for proper vision.

#### Other Animals

Outer pigment cells, the principle glia in the honey-bee (*Apis mellifera)* drone retina, spatially buffer K^+^ in the extracellular space around photoreceptors, based on electrophysiological studies (Coles, 1989). How this impacts animal vision is not yet established.

## Concluding Remarks

### Analogy in Glial Ionic Regulation Across Sensory Modalities

**FIGURE 3:**
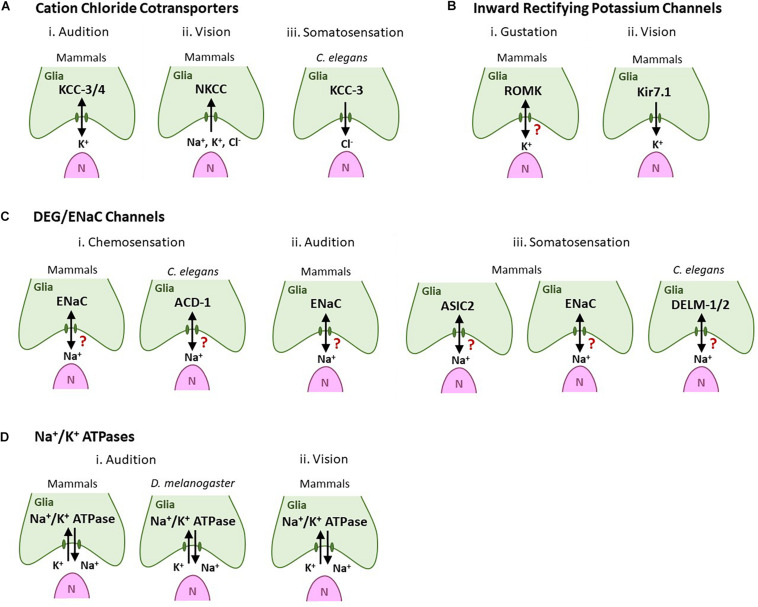
Glial ionic regulators regulate sensory neuron shape and functions across sensory modalities and species. Green cells are glia-like support cells. Magenta cells (N) are sensory cells. Arrows denote the direction of ion movement associated with sensation. A question mark denotes cases where the direction of ion movement is unknown. **(A)** Cation chloride cotransporters. (i) Audition: Glial KCC-3/KCC-4 in inner ear support cells regulates auditory transduction and their loss leads to hair cell degeneration and deafness. (ii) Vision: Mammalian RPE NKCC uptake K^+^, Na^+^, and Cl- to modulate the ionic milieu in the subretinal space. (iii) Somatosensation: Cl- extruded by glial KCC-3 regulates thermosensory NRE shape and animal behavior in *C. elegans*. **(B)** Inward rectifying K^+^ channels. (i) Type I taste cells express the ROMK channel, possibly to buffer extracellular K^+^. (ii) Mammalian Kir7.1 channels in RPE cells are implicated in spatial buffering of K^+^ for continued neuron excitability. **(C)** DEG/ENaC channels. (i) Chemosensation (olfaction and/or gustation): ENaC channels are expressed in vertebrate glia-like Type I taste cells (left). The glial DEG/ENaC channel ACD-1 regulates *C. elegans* chemotaxis behavior toward the odorant isoamyl alcohol and water-soluble tastants such as NaCl, acidic solutions, and lysine acetate (right). (ii) Audition: ENaC channels are expressed in support glia of the inner ear. (iii) Somatosensation: The DED/ENaC channel ASIC2 (left) and subunit ENaCβ (center) are expressed in glia-like cells associated with mammalian somatosensory receptor cells. *C. elegans* glial DELM-1/DELM-2 channels set basal sensory neuron excitability and drive sensitivity to mechanical stimuli (right). **(D)** Na^+^/K^+^ ATPases. (i) Audition: Na^+^/K^+^ ATPases are expressed in support inner ear cell in both mammals (left) and *Drosophila* (right) where they are required for audition. (ii) Vision: Na^+^/K^+^ ATPases are required in mammalian RPE cells to set the “dark current” required for vision.

One important glial function is regulation of ionic milieu around neurons (Olsen et al., 2015). It is evident that glia across sensory modalities, and species, deploy analogous machinery to regulate associated sensory cells/neurons. For example, many PNS glia across sensory systems and species use cation chloride cotransporters (CCCs), DEG/EnaC channels, inward rectifying channels and Na^+^/K^+^ ATPases to regulate K^+^, Na^+^, and Cl^–^ (above and Figure 3). However, PNS glia ionic regulation of NREs is an emerging field with many remaining unknowns. For example, the entire repertoire of ion channels and transporters and their subcellular localization is not described for any PNS glia. How any one glia regulates its ionic transporters/channels, and how this modifies sensorineural activity is not well-elucidated. Moreover, how these ionic regulators interact with each other is also poorly understood. Finally, it is not clear if all PNS glia within a sense organ are functionally and molecularly identical, even in regulating NRE ionic microenvironment. Thus, studies of such cross-utilized molecules in one modality may also inform on its function in another sensory context or species.

### Analogy in Glial Ionic Regulation Across CNS and PNS

Based on the literature, we also propose that studies of glial ionic regulation in the PNS may inform CNS-centric biology in two ways. First, we note that many neurological disorders also have a sensory component. For example, anosmia can precede motor deficits in Parkinson’s disease patients, and Autism patients exhibit significant sensory impairments (Marco et al., 2011; Doty, 2012). How these causally link across the PNS and CNS is not well-defined, and further investigation of sensory impairments in animal models where these ionic regulators are implicated may be insightful into early stages of disease-progression as well as diagnostics.

Second, comparative analyses of glial functions across CNS/PNS suggests that, despite deriving from distinct embryonic developmental cell lineages, glia across systems and species show partial functional and mechanistic analogy in the molecular strategies with which they regulate ionic milieu of associated neurons. Thus, CCC channels are implicated not only in many sensory systems (Figure 3A), but also in CNS disorders like epilepsy, hydrocephalus, autism, Anderman syndrome, and ischemic stroke (Garneau et al., 2017; Huang et al., 2019; Jin et al., 2019). Similarly, DEG/ENac channels express not across sensory systems, but are also implicated in forming hybrid channels with related ASIC channels to drive amiloride-sensitive currents and migration in human glioma cells (Kapoor et al., 2011; Sun et al., 2013). Inward rectifying potassium channels also not only regulate sensory glia biology (Figure 3B) but are also a prominent feature of mature post-mitotic astrocytes. They are implicated in many diseases including epilepsy, multiple sclerosis, glial malignancy (Olsen et al., 2015; Seifert et al., 2018). Lastly, while our compilation highlights a theme of sensory glia utilizing Na^+^/K^+^ ATPase (Figure 3D), these also are not a PNS-specific glia feature. They, along with many of the other channels above, are critical regulators of cerebral edema, and implicated in diseases like Alzheimer’s disease (Ugbode et al., 2017).

Furthermore, we note that this analogy in CNS and sense-organ glia functions persists beyond glial ionic regulation. For example, mammalian CNS astrocytes secrete thrombospondin (TSP)-1 and 2 to promote synaptogenesis (Christopherson et al., 2005). Analogously, the *C. elegans* sense-organ amphid sheath glia secretes the TSP-1 domain containing protein FIG-1 to modulate sensory neuron properties (Bacaj et al., 2008). Vertebrate astrocytes use the MEGF10 and MERTK phagocytic pathway to mediate synapse elimination (Chung et al., 2013), while *Drosophila* astrocytes similarly deploy the MEGF10 ortholog Draper to clear synaptic and neuronal debris following injury (MacDonald et al., 2006; Hilu-Dadia and Kurant, 2020; Raiders et al., 2021b) and *C. elegans* peripheral sense organ glia use a similar (albeit not identical) machinery to regulate sensory neuron shape and animal behavior (Raiders et al., 2021a).

We therefore propose that investigations into PNS glia biology may be broadly relevant. Comparative studies across CNS/PNS, and species, may inform on novel insights into ontogenic and evolutionarily conserved molecular mechanisms by which glia sensorineural health and disease.

## Author Contributions

SR and AS conceptualized and co-wrote the manuscript. Both authors contributed to the article and approved the submitted version.

## Conflict of Interest

The authors declare that the research was conducted in the absence of any commercial or financial relationships that could be construed as a potential conflict of interest.

## Publisher’s Note

All claims expressed in this article are solely those of the authors and do not necessarily represent those of their affiliated organizations, or those of the publisher, the editors and the reviewers. Any product that may be evaluated in this article, or claim that may be made by its manufacturer, is not guaranteed or endorsed by the publisher.
